# COVID-19 testing, infection, and vaccination among deported Mexican migrants: Results from a survey on the Mexico-U.S. border

**DOI:** 10.3389/fpubh.2022.928385

**Published:** 2022-07-29

**Authors:** Ana P. Martínez-Donate, Catalina Correa-Salazar, Leah Bakely, Jesús Eduardo González-Fagoaga, Ahmed Asadi-Gonzalez, Mariana Lazo, Emilio Parrado, Xiao Zhang, Maria Gudelia Rangel Gomez

**Affiliations:** ^1^Department of Community Health and Prevention, Dornsife School of Public Health, Drexel University, Philadelphia, PA, United States; ^2^Mexico Section of the U.S.-Mexico Border Health Commission, Tijuana, Mexico; ^3^School of Medicine and Psychology, Autonomous University of Baja California, Tijuana, Mexico; ^4^Department of Sociology, University of Pennsylvania, Philadelphia, PA, United States; ^5^Department of Pediatrics, School of Medicine and Public Health, University of Wisconsin, Madison, WI, United States; ^6^El Colegio de la Frontera Norte, Tijuana, Mexico

**Keywords:** COVID-19 infection, COVID-19 testing, COVID-19 vaccination, Mexican migrants, detention, deportation, Latino health, U.S.-Mexico border region

## Abstract

**Background:**

Migrants detained and held in immigration and other detention settings in the U.S. have faced increased risk of COVID-19 infection, but data on this population is scarce. This study sought to estimate rates of COVID-19 testing, infection, care seeking, and vaccination among Mexican migrants detained by U.S. immigration authorities and forcibly returned to Mexico.

**Methods:**

We conducted a cross-sectional probability survey of Mexican migrants deported from the U.S. to three Mexican border cities: Tijuana, Ciudad Juárez, and Matamoros (*N* = 306). Deported migrants were recruited at Mexican migration facilities after being processed and cleared for departure. A two-stage sampling strategy was used. Within each city, a selection of days and shifts were selected during the operating hours of these deportation facilities. The probability of selection was proportional to the volume of migrants deported on each day of the month and during each time period. During the selected survey shifts, migrants were consecutively approached, screened for eligibility, and invited to participate in the survey. Survey measures included self-reported history of COVID-19 testing, infection, care seeking, vaccination, intentions to vaccinate, and other prevention and risk factors. Weighted data were used to estimate population-level prevalence rates. Bivariate tests and adjusted logistic regression models were estimated to identify associations between these COVID-19 outcomes and demographic, migration, and contextual factors.

**Results:**

About 84.1% of migrants were tested for COVID-19, close to a third were estimated to have been infected, and, among them, 63% had sought care for COVID-19. An estimated 70.1% had been vaccinated against COVID-19 and, among those not yet vaccinated, 32.5% intended to get vaccinated. Close to half (44.3%) of respondents had experienced crowdedness while in detention in the U.S. Socio-demographic (e.g. age, education, English fluency) and migration-related (e.g. type of detention facility and time in detention) variables were significantly associated with COVID-19 testing, infection, care seeking, and vaccination history. Age, English fluency, and length of detention were positively associated with testing and vaccination history, whereas detention in an immigration center and length of time living in the U.S. were negatively related to testing, infection, and vaccination history. Survey city and survey quarter also showed adjusted associations with testing, infection, and vaccination history, reflecting potential variations in access to services across geographic regions and over time as the pandemic unfolded.

**Conclusion:**

These findings are evidence of increased risk of COVID-19 infection, insufficient access to testing and treatment, and missed opportunities for vaccination among Mexican migrants detained in and deported from the U.S. Deportee receiving stations can be leveraged to reduce disparities in testing and vaccination for deported migrants. In addition, decarceration of migrants and other measures informed by public health principles must be implemented to reduce COVID-19 risk and increase access to prevention, diagnostic, and treatment services among this underserved population.

## Introduction

According to the 2021 Census, 18% of the United States (U.S.) population is Latino/Hispanic, and one of every three Latinos is foreign-born (1). Migration status constitutes a social determinant of health (2, 3) because of its relation to stigma, language (4), lower social and economic status, cultural barriers (5, 6), and legal status. Among Latino immigrants, two in three are not U.S. citizens and around eight million are unauthorized immigrants (7). Furthermore, 64% are not proficient in English (8). Data from around the world has shown a disproportionate impact of the COVID-19 pandemic on migrants (9). In the U.S., while availability of disaggregated data by nativity has been limited (10), there is mounting evidence that foreign-born Latinos have borne a heavy burden of the COVID-19 pandemic and have experienced significant barriers to testing and treatment (2, 10, 11).

For Latino immigrants, increased COVID-19 risk is related to social and structural determinants of health (12–15). These include occupational profile (2, 10), such as inability to work remotely, lack of sustainable and safe working conditions (10, 16), overrepresentation in “essential” frontline jobs (2, 10, 17), lack of paid leave if they get sick, (16) lack of flexible working hours, lack of leisure time (12), lack of health insurance or healthcare through work, (10, 18) and/or ability to miss work (19). Other risk factors are limited healthcare access and insurance rates (20, 21), overcrowding, (11, 22, 23) immigration enforcement (3, 10, 11, 24–26), and lack of legal protections (27). Latino immigrants in essential occupations had the highest risk of excess death during the pandemic among the working-age group (10, 17).

A central mechanism of both increased exposure to COVID-19 and COVID-19 infection among Latino immigrants is the U.S.' persecution, detention, and deportation system, which is reinforced by anti-immigration policies, institutional rhetoric and racial profiling. Between 2015 and 2018, migrant detentions and deportations increased by over 30 and 13%, respectively (24). Males from Mexico, Guatemala, El Salvador and Honduras account for 84–89% of Immigration and Customs Enforcement's (ICE) detentions (24) and 90–94% of deportations. Mexicans, in particular, account for the highest proportion of yearly deportations. The Trump administration used the pandemic as an excuse to infringe further upon the rights of immigrants in the U.S. (28) and enforce indiscriminate detention and deportation policies. (29, 30) Throughout the pandemic, ICE has deported detainees who have tested positive for COVID-19 in detention centers in hotspot states like Texas, Arizona, California, and Florida to Mexico, Honduras, Guatemala, and El Salvador (2). According to Reuters, through December 2021, Guatemalan migrants alone were deported on at least 184 flights (31). There is also some evidence that U.S. deportation policies have resulted in the spread of COVID-19 among migrant sending countries including Mexico, Central America, and Haiti (32).

Detention and deportation centers pose significant risk of exposure and transmission of COVID-19 for migrants because they are forced to be in close proximity to others, they are confined to enclosed spaces and they have limited access to testing (33). Evidence drawing from ICE detention center data found that, during the pandemic, between 70 and 90% of all detainees had a risk of becoming infected due to the conditions in which they were held (34). ICE has publicly confirmed that testing and releases based on pre-existing health conditions have been rare. (33) Despite these alarming data, there has been insufficient research on COVID-19 risk, infection rates, and access to vaccination among foreign-born Latinos in the U.S., including those in detention or deported to their countries of origin. Evidence that suggests that COVID-19 has disproportionately impacted migrant, ethnic, and racial minorities in the U.S. has rarely been disaggregated by nativity and has seldom explored the association between sociodemographic, migration, and other contextual characteristics and COVID-19 related outcomes. There is a lack of data available for undocumented foreign-born Latinos because they are hardly included in research or public health surveillance and, when they are included, their legal status is not known (25, 35).

This study addresses this research gap by examining COVID-19 testing, infection, treatment and vaccination rates among Mexican migrants deported from the U.S. Using data from Project Migrante, we estimate COVID-19 testing, infection, treatment, and vaccination rates among Mexican immigrants deported from the U.S. We also explore demographic, migration, and contextual factors associated with differential rates of infection and access to services among this vulnerable population.

## Materials and methods

### Study design

This study uses data from Project Migrante, an observatory on migrant health on the Mexico – U.S. border. Since 2009, Migrante has conducted a series of cross-sectional health surveys of Mexican migrant flows traveling through this region. The surveys for this study were administered in the Mexican border cities of Tijuana, Matamoros, and Ciudad Juárez. These cities were selected because their locations in the Western, Central, and Eastern regions of the Mexico-U.S. border and transportation infrastructure uniquely position them to receive flows from and to a variety of sending and receiving regions in the U.S., Mexico, and other Latin American countries. Moreover, they receive the flows of Central American ‘returnee' migrants that are either sent back to Mexico to wait for review of their refugee applications under Title 42 or are simply expelled from the United States.

Migrante relies on a two-stage sampling design with two dimensions (time and space) to sample Mexican migrants from three different migration flows: Southbound migrants traveling from the Mexican border to other areas of Mexico after a stay in the U.S. or the Mexican border region; Northbound migrants arriving at the Mexican border as their final destination or in transit to the U.S.; and migrants deported by U.S. migration authorities and released at the Mexican border by the National Migration Institute of Mexico (INM, per its acronym in Spanish). The sample for this study comes from a Migrante survey that sampled migrants from the deported migrant flow between August 2020 and July 2021. Data was collected in Tijuana from August 26th, 2020 through June 28th, 2021, in Matamoros from November 6th, 2020 to July 30th, 2021, and in Ciudad Juárez from February 9th, 2021 to April 27th, 2021. For the sampling of deported migrants, we used a two-stage probability sampling strategy to estimate the characteristics of the individuals returned to Mexico through the study repatriation points. A sampling frame was elaborated considering two axes: time and space. The time axis was defined as the number of calendar days in the month that can be 28, 30 and 31, or even less because there are days in which there was no flow (i.e., US Holidays); in addition, each day was divided into shifts, according to the particular dynamics of the deportation station of each city. The second axis -space- was defined based on the location of Mexican immigration authorities' offices where migrants are repatriated by their US counterparts. Project Migrante selected Tijuana, Ciudad Juárez and Matamoros, as these cities account for a significant proportion of the repatriations of Mexican migrants (between January and April 2022, these three cities concentrated 47% of total repatriations, according to data from the Mexican Migration Institute). The combination of all possible sampling places and times represents the entire sampling frame from which specific sampling shifts were selected. The first stage of the sampling process consisted of determining the time and place of the survey shifts for the next observation period (i.e., usually the next month). The number of survey shifts for each month was selected a priori based on budgetary considerations as well as knowledge about the behavior of the flow. The next step was to assign the number of sampling shifts to each of the strata (i.e., days and shifts) in which it was known that there would be repatriations of Mexican migrants during the next sampling period. The assignment was conducted according to the weight represented by each shift within the day.

The second stage of sampling pertained to the selection of the specific units of observation (i.e. migrants) during the selected survey shifts. For the deported flow, the survey was conducted in the hallways through which deportees exit the deportation stations of each of the study cities. As they were cleared for departure by Mexican migration authorities, migrants were consecutively approached by trained Mexican research assistants and screened for eligibility to determine if they belonged into the target population. Eligibility criteria included being an adult age 18 or older, being fluent in Spanish, and having just been deported from the U.S. and processed by Mexican migration authorities at the sampling point. By design, all migrants deported through these stations were Mexican nationals. Persons who were unable to answer questions due to mental or physical limitations were also excluded from the study. Eligible individuals were invited to provide informed consent to participate in the survey. Consenting individuals completed a questionnaire designed to characterize their health and access to health services. No names or identifying information were collected from survey participants (36).

### Study measures

Migrante surveys consist of an interviewer-administered questionnaire and several biometric tests. The questionnaire collects information on demographics, socio-ecological health determinants, and a detailed migration and deportation history. There are also questions on health outcomes and healthcare access related to the different focus areas of each survey wave (e.g. HIV and other sexually transmitted infections, non-communicable chronic disease, etc.). Biometric rapid tests are used to screen for infectious diseases (e.g., HIV, syphilis, etc.), risk factors for cardiovascular disease (e.g., blood glucose, cholesterol levels, etc.), or stress levels (i.e., cortisol), depending on the survey wave.

Starting in August of 2020, questions about COVID-19 were added to the Migrante questionnaires.

COVID-19 questions queried about having ever been tested for COVID-19 (yes/no), having tested positive for COVID-19, having been told by a doctor or other health care provider they had COVID-19, or believing they had been infected with COVID-19. A positive response to any of these last three questions was used as an indication of COVID-19 infection history. Those with a positive test, doctor diagnosis, or a belief they had been infected with COVID were further asked whether they had sought treatment for COVID-19 (yes/no). Additional questions queried about COVID-19 vaccination history (yes/no), intentions to get vaccinated in the future among those not yet vaccinated (yes/no/don't know), reasons for not intending to get vaccinated among those unsure or not planning to get vaccinated (e.g. fear of side effects, not knowing where to obtain a vaccine, not believing in vaccines, etc.). A final set of questions asked about other preventive and risk behaviors, including mask wearing, hand washing, staying home, ability to work from home, and exposure to crowded conditions while in detention in the U.S. (yes/no). Questions regarding country where testing, infection, treatment, and vaccination took place were also included in this study.

Socio-demographic measures included age (in years), gender, indigenous ethnic identity (whether the respondent self-identified as member of an indigenous Mexican population), marital status (recoded as married/living with a partner vs. other), and education level (recoded as less than high school vs. high school or higher level). English fluency level was assessed with a four-point Likert-scale from Not speaking English at all to Speaking English Very Well.

Migration measures included previous history of migration to the U.S. (yes/no), last country of residence (the U.S., Mexico, other), length of residence in the U.S. (in years), previous history of deportation (yes/no), time spent in detention in the U.S. (in days), type of detention facility where participants were held in the U.S. (recoded as immigration detention center versus other type of detention facility), and most recent immigration status in the U.S. (recoded as unauthorized vs. other).

Contextual variables included city in which the respondent was recruited (Tijuana, Ciudad Juárez, or Matamoros) and quarter during which they completed the survey. Because migrants detained in different regions may have been held in different detention facilities and deported through different border cities, the recruitment city was thought of as potential proxy for geographic variations in COVID-19 risk and access to testing and vaccination. Infection rates and availability of testing and vaccination varied throughout the pandemic. Survey quarter was used to capture these potential time variations.

### Statistical analyses

Survey weights (i.e. expansion factors) were computed for each observation to obtain parameter estimates for the deported migrant flow. Our weighting procedures were modeled after those used in previous Migrante Project phases (36). The formula for the calculation of the expansion factors relied on the notion that the final survey shifts were selected from the universe of strata defined from the combination of the components of the temporal and spatial axes. Each of the components of these axes has a probability of selection based on the sampling frame. In the case of the temporal axis, there was the number of days of the month in which it was known that there would be a flow of repatriated migrants, as well as the weight represented by each of the shifts in which a day was divided. In the case of the spatial axis, there was only one repatriation point per city (which are independent) and within these cities there was only one exit door, so the weight of each city was equal to one. In addition, we knew the specific size of the flow of repatriated migrants through the study cities during the survey period, since the immigration authorities keep a record of that number. Thus, the expansion factor for each stratum was calculated from:


$$\begin{array}{l} W_{jk}=\frac{{\frac{P_{ij}}{S_{jk}}}^{*}D_{k}}{Q_{jk}} \end{array}$$

where

W_jk_ is the statistical weight to estimate the total number of migrants in the j^th^ stratum of the k^th^ month
P_ij_ is the number of migrants repatriated during S_k_ of the j^th^ stratum (this information is obtained from Mexican migration authorities)
S_jk_ is the number of days assigned to the j^th^ stratum and worked during the k^th^ month
D_k_ is the number of days available to select during the k^th^ month
Q_jk_ is the number of migrants with a complete questionnaire in the j^th^ stratum during the k^th^ month

After weighting the data with the expansion factors obtained from the formula above, we estimated descriptive statistics of socio-demographic characteristics, migration history, and contextual variables. Descriptive statistics were also run to estimate prevalence of COVID-19 testing, infection history, treatment, vaccination and other preventive and risk factors.

Using unweighted data, we also examined bivariate associations between COVID-19 outcomes and socio-demographics, migration, and contextual variables. In addition, we estimated adjusted logistic regression models to examine independent associations between each demographic, migration, and contextual variable, on the one hand, and COVID-19 testing, infection, care, and vaccination status, on the other, adjusted for age, gender, education, ethnicity, and marital status.

## Results

### Population characteristics

The target sample size was 300 deported migrants, 100 from each study site. Sample size and statistical power calculations were based on sexual and reproductive health outcomes (e.g., multiple sex partners, lifetime sexually transmitted infections), which represented the main focus of the survey when it was designed, prior to the pandemic. The final survey sample included 306 deported migrants (response rate =76%), including 127 from Tijuana, 155 from Matamoros, and 24 from Ciudad Juarez. Changes in both the volume of migrants deported through Ciudad Juarez and in the times during which the deportation station in this city was operating made it impossible for our team to achieve the desired sample size of 100. As a result, we oversampled participants in Tijuana and Matamoros to reach our target sample size of 300. Among the final sample, 34 participants identified as females and 272 as males. A comparison of participants who completed the survey vs. eligible individuals who did not complete the survey indicated that the two groups did not differ significantly in terms of their gender, marital status, education, race/ethnicity, or country of residence. However, survey respondents were slightly younger (average age was 38.3 vs. 39.7 years, Odds Ratio [OR] = 0.97, *p* = 0.006), less likely to be deported through Tijuana (OR = 0.46, *p* = 0.001) or Ciudad Juarez (OR = 0.24, *p* = 0.001), and more likely to be deported through Matamoros (OR = 7.01, *p* = 0.001), compared to non-respondents.

Based on the calculated expansion factors and the information supplied by the Mexican Migration Institute, survey participants (N = 306) represented a total weighted population of 14,841 Mexican migrants deported through Tijuana, Ciudad Juarez, and Matamoros during the survey period. The weighted distribution by city was as follows: 73.9% deported through Tijuana, 22.4% through Matamoros, and 3.7% through Ciudad Juárez (Table 1). Weighted descriptive analyses indicated that deported migrants were 38-years-old on average (standard deviation [SD] = 10.5). Most (92%) were male. Less than a quarter (22.2%) had completed high school. Approximately 5.4% identified as members of an indigenous community. In terms of migration history, 92.1% had a history of migration to the U.S. and 85.8% reported the U.S. was their most recent country of residence. Average time living in the U.S. was 17.3 years (SD = 12.0) and, within the last 12 months, the average deported migrant had spent 9.7 months in the U.S. (SD = 4.5). More than half of the migrants had a previous history of deportation (57.2%), and the majority (87.4%) had an unauthorized immigration status in the U.S. Even so, the majority was employed (63% full time and 16.8% part time) or self-employed (5.9%).

**Table 1:** Descriptive statistics of selected socio-demographics characteristics and migration history for migrants deported from the U.S. to Tijuana, Matamoros and Ciudad Juárez, Mexico, between August 2020 and July 2021 (weighted population=14,841).

	**%**	**Mean**	**SD**
**Socio-demographics**			
Age (years)		38.3	10.5
Gender (male)	91.9		
Education level (high school or more)	22.2		
Marital status (married or living with a partner)	36.7		
Ethnic or racial minority^1^	5.4		
English fluency			
Not at all	11.9		
Not very well	42.1		
Well	22.4		
Very well	23.6		
**Migration history**			
Previous history of migration to U.S.	92.1		
Most recent country of residence is the U.S.	85.8		
Lifetime length of residence in the U.S. (years)^2^		17.3	12.0
History of deportation (1+ times before the most recent event)	57.2		
Unauthorized immigration status in the U.S.^3^	87.4		
Length of detention in the U.S. (months)		17.2	40.0
**Survey context**			
Survey location			
Tijuana	73.9		
Matamoros	22.4		
Ciudad Juárez	3.7		
Survey quarter			
First quarter (Aug '20 - Oct '20)	17.2		
Second quarter (Nov '20 - Jan '21)	20.7		
Third quarter (Feb '21 - Apr '21)	7.2		
Fourth quarter (May '21 - Jul '21)	54.9		

1 Combined variable for anyone who described themselves as indigenous and/or of African descent.

2 Only asked to participants who reported U.S. migration experience.

3 Only asked to people who were in the U.S. for at least 30 days in last 12 months and were not detained for the entirety of their time in the U.S.

### COVID-19 testing

Most deported migrants (84.1%) had been tested for COVID-19 at least once since the start of the pandemic, with the majority of them reporting testing in the U.S. (94.3%; Table 2). For those tested in the U.S., 79.1% were tested in an immigration detention center or prison; 16.9% were tested outside in the community by a healthcare provider; 2.1% in urgent care; 1.0% in a hospital or emergency room; and 0.9% in a local health department testing site. In contrast, those tested in Mexico were tested mostly at a health center or hospital (93.1%) and 45.7% were tested in other settings. Testing locations were not mutually exclusive.

**Table 2:** Descriptive statistics of prevalence of COVID-19 testing, diagnosis, infection, treatment, and vaccination among migrants deported from the U.S. to Tijuana, Matamoros and Ciudad Juárez, Mexico, between August 2020 and July 2021 (weighted population=14,841).

	**%**
**COVID-19 lifetime testing**	
Ever tested for COVID-19	84.1
Testing location (among those tested)^1^	
U.S.	94.3
Mexico	5.2
Other country	0.2
**COVID-19 lifetime prevalence** ^1^	
Ever had a positive COVID-19 test result	12.1
Ever diagnosed with COVID-19 by a health care professional	5.5
Think they have ever had COVID-19	16.2
Overall COVID lifetime prevalence^2^	30.4
**COVID-19 diagnosis and care**	
Diagnosis location^1^	
U.S.	94.3
Mexico	5.7
Care sought for COVID-19 care (among those who had COVID-19)	63.0
Care location^1^	
U.S.	98.7
Mexico	7.3
Type of location - U.S.^1^	
Immigration detention center or prison	79.1
Primary care or doctor's office	16.9
Urgent care facility	2.1
Hospital or emergency room	1.0
Local health department	0.9
Type of location – Mexico^1^	
SSA Health center or hospital	93.1
Other	45.7
**COVID-19 vaccination** ^**3**^	
Vaccinated (at least one dose)	70.1
Vaccination location (among those vaccinated)^1^	
U.S.	99.9
Mexico	0.2
Intends to get vaccinated (among those not vaccinated)	
Yes	32.5
No	41.0
Don't know	26.5
Reasons for vaccine hesitancy^3^^,^^4^	
Concern about side effects	74.0
Don't know where to get vaccinated	13.0
Don't believe in vaccines	4.8
Concern about data collected at vaccine sites	2.0
Mistrust of doctors	0.6
Other	2.4
Don't know/refuse to answer	3.2
**Prevalence of other preventive and risk factors** ^**1**^^**,**^^**3**^	
Mask wearing (last 7 days)	98.1
Frequent hand washing or sanitizing (last 7 days)	96.3
Staying home most of the time (last 7 days)	62.6
Ability to work from home (last 7 days)	0.4
Experienced crowdedness while in detention in the U.S.	44.3

1 Answers are not mutually exclusive.

2 Combining positive result, diagnosis by a healthcare provider, and/or think they have had COVID-19.

3 Weighted population for questions about vaccination is 9,285 because they were not added to the survey until January 29th, 2020.

4 Asked to participants who said they would not get vaccinated or they didn't know if they would get vaccinated.

Results from bivariate analyses indicated that testing varied significantly by marital status, self-reported indigenous identity, and level of English fluency. In general, testing was more likely to be reported by non-married migrants (Chi square = 16.74, *p* < 0.001), migrants who did not identify as members of an indigenous group (Chi square =4.79, *p* = 0.029), and those with higher levels of English fluency (Linear-by-Linear Association =6.394, *p* = 0.011). Based on migration history, migrants with a history of U.S. migration (Chi square =24.89, *p* < 0.001), those whose residence was in the U.S. (Chi square =76.5, *p* < 0.001), and migrants who had never before been deported (Chi square =5.93, *p* = 0.015) were more likely to report testing experience. In general, testing rates and time in the U.S. showed a U-shape association (Chi square = 37.6, *p* < 0.001), with higher rates among new migrants (0–2 years in the U.S.) and those who had been in the U.S. for 21 years or more. Testing rates were lower for migrants detained in immigration facilities (Chi square =33.69, *p* < 0.001) and, in general, increased by length of time in detention (Linear-by-linear association = 69.2, *p* < 0.001).

Bivariate analyses showed that testing rates also varied significantly by survey site, with the highest testing rates estimated for migrants deported through Ciudad Juárez (Chi square = 23.33, *p* < 0.001), and increasing by quarter (Linear-by-linear association =22.67, *p* < 0.001) (Table 3). These variations by study site and survey quarter may reflect geographic and time variations in availability of testing services.

**Table 3:** Bivariate associations between COVID-19 outcomes and demographic, migration, and contextual variables among migrants deported from the U.S. to Tijuana, Matamoros and Ciudad Juárez, Mexico, between August 2020 and July 2021 (weighted population=14,841).

	**Tested for COVID-19**			**COVID-19 infection**			**Care-seeking**			**Vaccinated**		
	**Yes** **%**	**No** **%**	***p*** ^*^	**Yes** **%**	**No** **%**	***p*** ^*^	**Yes** **%**	**No** **%**	***p*** ^*^	**Yes** **%**	**No** **%**	***p*** ^*^
Age (yrs)			0.284			0.807			**0.006**			**0.017**
18–29	69.0	31.0		10.3	89.7		44.4	55.6		30.4	69.6	
30–36	76.9	23.1		8.9	91.1		57.1	42.9		41.4	58.6	
37–45	74.7	25.3		1.2	98.8		90	10.0		61.9	38.1	
>45	82.8	17.2		13.8	86.2		12.5	87.5		60.9	39.1	
Gender			0.824			0.397			1.00			0.717
Male	75.3	24.7		11.8	88.2		53.1	46.9		48.9	51.1	
Female	73.5	26.5		5.9	94.1		50.0	50.0		37.5	62.5	
Education			0.194			**0.042**			0.429			0.649
Less than high school	73.3	26.7		9.1	90.9		47.6	52.4		49.3	50.7	
High school or more	80.8	19.2		17.6	82.4		61.5	38.5		44.0	56.0	0.629
Marital status			**<0.001**			0.817			0.774			
Married	62.5	37.5		11.7	88.3		50.0	50.0		45.0	55.0	1.00
Single/never married/other	83.2	16.8		10.8	89.2		55.0	45.0		50.0	50.0	
Ethnic or racial minority			**0.029**			0.297			1.00			
Yes	56.5	43.5		17.4	82.6		50.0	50.0		40.0	60.0	
No	77.0	23.0		10.4	89.6		51.7	48.3		48.4	51.6	
English fluency			**0.011**			**0.028**						**0.037**
Not at all	81.4	18.6		5.7	94.3		100	0.0	0.028	45.0	55.0	
Not very well	86.7	13.3		14.7	85.3		27.3	72.7		48.1	51.9	
Well	92.6	7.4		14.8	85.2		50.0	50.0		40.9	59.1	
Very well	97.1	2.9		20.0	80.0		71.4	28.6		82.4	17.6	
Previous history of migration to U.S.			**<0.001**			0.28			1.00			1.00
No	42.1	57.9		5.3	94.7		50.0	50.0		0.0	100	
Yes	79.6	20.4		12.0	88.0		53.1	46.9		100	0.0	
Time in the U.S. (yrs)			**<0.001**			**0.04**			0.317			**<0.001**
0–2	84.8	15.2		20.0	80.0		37.5	62.5		80.0	20.0	
3–10	50.0	50.0		6.6	93.4		60.0	40.0		11.8	88.2	
11–21	73.9	26.1		7.2	92.8		80.0	20.0		40.0	60.0	
>21	89.5	10.5		10.5	89.5		62.5	37.5		46.9	53.1	
Most recent residence was U.S.			**<0.001**			0.434			0.681			0.074
No	38.8	61.2		8.8	91.2		42.9	57.1		23.1	76.9	
Yes	88.0	12.0		11.9	91.2		55.6	44.4		51.8	48.2	
Previous history of deportation			**0.015**			0.637			0.755			0.216
No	84.7	15.3		11.4	88.6		31.3	68.7		55.3	44.7	
Yes	72.4	27.6		13.3	86.7		21.9	78.1		42.6	57.4	
Time detained (days)			**<0.001**			**0.008**			**0.081**			**0.014**
0–3	39.5	60.5		10.5	89.5		33.3	66.7		29.4	70.6	
4–62	84.0	16.0		2.7	97.3		0.0	100		43.8	56.2	
63–365	92.2	7.8		7.8	92.2		17.6	82.4		33.3	66.7	
>365	95.8	4.2		23.6	76.4		64.7	35.3		63.6	36.4	
Detention facility			**<0.001**			**0.008**			0.429			**0.026**
Other	94.1	5.9		17.6	82.4		47.6	52.4		58.8	41.2	
Immigration center	64.9	35.1		7.6	92.4		61.5	38.5		35.7	64.3	
Unauthorized immigration status			0.31			**<0.001**			1.00			**0.049**
No	88.0	12.0		34.6	65.4		55.6	44.4		73.3	26.7	
Yes	77.3	22.7		9.8	90.2		52.0	48.0		43.8	56.2	
Survey location			**<0.001**			**<0.001**			0.534			**0.002**
Tijuana	61.9	38.1		18.1	81.9		47.8	52.2		73.7	26.3	
Matamoros	82.6	17.4		2.6	97.4		50.0	50.0		50.9	49.1	
Ciudad Juárez	95.8	4.2		29.2	70.8		71.4	28.6		20.8	79.2	
Survey quarter			**<0.001**			**0.027**			**0.066**			**<0.001**
First quarter (Aug '20 - Oct '20)	56.2	43.8		12.2	87.8		54.4	45.6		NA	NA	
Second quarter (Nov '20 - Jan '21)	77.8	22.2		3.2	96.8		0.0	100		0.0	100	
Third quarter (Feb '21 - Apr '21)	92.9	7.1		25.0	75.0		71.4	28.6		17.9	82.1	
Fourth quarter (May '21 - Jul '21)	88.7	11.3		19.4	80.6		58.3	41.7		67.2	32.8	

* p-values based on bivariate Chi-square tests.

NA, Not applicable. Vaccination questions were added in the second quarter of the survey.

Bold values indicate p value was less than 0.05.

After adjusting for gender, age, ethnic identity, education, and marital status, most variables remained significantly associated with testing history, including age (i.e., being older than 45 vs. 18–29), marital status, English fluency, having a history of migration to the U.S., having a residence in the U.S., length of residence in the U.S., time in detention, type of facility where migrants were detained, survey city, and survey quarter (Table 4).

**Table 4:** Adjusted associations between COVID-19 outcomes^1^ and demographic, migration, and contextual variables among migrants deported from the U.S. to Tijuana, Matamoros and Ciudad Juárez, Mexico, between August 2020 and July 2021 (weighted population=14,841).

	**Tested for COVID-19**			**COVID-19 infection**			**Vaccinated**		
	**AOR** ^2^	***p***		**AOR** ^2^	**p**		**AOR** ^2^	***p***	
Age (yrs)									
18–29	1			1			1		
30–36	1.57	0.225		0.88	0.806		1.75	0.355	
37–45	1.39	0.367		1.12	0.827		3.92	**0.035**	
>45	2.87	**0.021**		1.7	0.323		4.07	**0.03**	
Gender									
Male	1			1			1		
Female	0.711	0.443		0.417	0.251		0.631	0.565	
Education									
Less than high school	1			1			1		
High school or more	1.83	0.087		2.53	**0.02**		0.919	0.867	
Marital status									
Married	1			1			1		
Single/never married/other	2.99	**<0.001**		0.924	0.838		1.23	0.649	
Ethnic or racial minority^1^									
No	1			1			1		
Yes	0.413	0.064		1.53	0.484		0.507	0.5	
English fluency									
Not at all	1			1			1		
Not very well	1.39	0.495		2.5	0.148		1.23	0.764	
Well	2.43	0.161		2.22	0.242		1.06	0.942	
Very well	7.82	0.057		3.69	0.061		6.59	**0.027**	
Previous history of migration to U.S.									
No	1			1			NE		
Yes	4.4	**<0.001**		2.42	0.265				
Time in the U.S. (yrs)									
0–2	1			1			1		
3–10	0.2	**<0.001**		0.32	**0.049**		0.022	**<0.001**	
11–21	0.616	0.296		0.395	0.113		0.14	**0.022**	
>21	1.59	0.376		0.61	0.333		0.146	**0.025**	
Most recent residence - U.S.									
No	1			1			1		
Yes	10.52	**<0.001**		1.53	0.375		3.28	0.109	
Previous history of deportation									
No	1			1			1		
Yes	0.548	0.08		1.14	0.74		0.438	0.08	
Time detained (days)									
0–3	1			1			1		
4–62	7.24	**<0.001**		0.24	0.081		1.53	0.595	
63–365	15.69	**<0.001**		0.51	0.300		1.37	0.681	
>365	32.42	**<0.001**		3.04	**0.028**		3.89	**0.044**	
Detention facility									
Other facility	1			1			1		
Immigration center	0.118	**<0.001**		0.396	**0.019**		0.264	**0.01**	
Unauthorized immigration status^3^									
No	1			1			1		
Yes	0.341	0.121		0.148	**<0.001**		0.379	0.174	
Survey location									
Tijuana	1			1			1		
Matamoros	2.51	**0.003**		0.1	**<0.001**		0.653	0.514	
Ciudad Juárez	15.41	**0.011**		2.24	0.137		0.076	**0.001**	
Survey quarter									
First quarter (Aug '20 - Oct '20)	1			1			NE	NE	
Second quarter (Nov '20 - Jan '21)	2.3	**0.012**		0.246	**0.027**				
Third quarter (Feb '21 - Apr '21)	11.02	**0.002**		2.79	**0.077**				
Fourth quarter (May '21 - Jul '21)	5.45	**<0.001**		1.83	0.226				

1 Adjusted regression models could not be estimated for care seeking due to the small number of migrants for whom this question was applicable.

2 Adjusted odds ratios (AOR) based on logistic regression models adjusted for age, gender, education, marital status, ethnic identity.

NE, Not estimated. Adjusted associations could not be calculated because of small size of subsample or lack of variation within some categories.

Bold values indicated p values were less than 0.05.

### COVID-19 infection

An estimated 12.1% of migrants who had ever tested for COVID-19 had a positive result, which indicated COVID-19 infection. In addition, regardless of testing history, 16.2% thought they had been infected with COVID-19 and 5.5% had a doctor diagnosis. Combining these three different indicators, we estimated that 30.4% of deported migrants had a history of COVID-19 infection (Table 2). Among migrants diagnosed with COVID-19, 94.3% were diagnosed in the U.S. and 5.7% in Mexico. Based on bivariate analyses, rates of COVID-19 infection history (i.e. based on a positive test result, a doctor diagnosis, and/or their belief that they had had COVID-19) varied by demographic, migration, and survey variables. In general, infection rates were higher for migrants who had graduated from high school (Chi square =4.12, *p* = 0.042), migrants with higher levels of English fluency (Linear-by-linear association =4.85, *p* = 0.028), migrants with shorter lengths of residence in the U.S. (0–2 years in the U.S., Likelihood Ratio =8.33, *p* = 0.04), migrants with longer periods spent in detention (Linear-by-linear association =7.04, *p* = 0.008), migrants detained in facilities other than immigration centers (Chi square =6.94, *p* = 0.008), and authorized migrants (Chi square =25.6, *p* < 0.001). Infection rates also varied significantly by survey location (Likelihood Ratio =25.6, *p* < 0.001) and increased over the course of the survey (Linear-by-linear association =4.91, *p* = 0.027; Table 3).

After adjustment for age, gender, ethnic identity, marital status, and education, the variables that remained significantly associated with infection history in regression models were level of education, time living in the U.S., time spent in detention, type of detention facility, unauthorized status, survey city, and survey quarter (Table 4).

### COVID-19 care seeking

An estimated 27.0% of all deported migrants had sought care for COVID-19 at some point; this percent increased to 63.0% when restricted to those who had tested positive, been told by a doctor, and/or believed they had had COVID-19. Among migrants who sought care for COVID-19, 98.7% did so in the U.S. and 7.3% did so in Mexico. In the U.S., migrants sought care most frequently in immigration or other detention centers (79.1%), followed by doctors' offices (16.9%). About 2.1% sought care in an urgent care center and 1% in a hospital or emergency room. In Mexico, migrants sought care most frequently at a health center or hospital (93.1%). Country and location of care were not mutually exclusive, and some migrants sought care in more than one country and/or location within the country (Table 2).

Bivariate analyses showed only age was significantly associated with COVID-19 care-seeking (Likelihood Ratio =12.56, *p* = 0.006). Care-seeking rates increased by age group until age 45 and were lowest for migrants over 45 years old. In addition, we found marginally significant associations between care-seeking and time in detention (Linear-by-linear Association =3.04, *p* = 0.081), suggesting care-seeking increased as time detained increased. We also found marginally significant associations between care-seeking and survey quarter (Likelihood Ratio =7.18, *p* = 0.066), with the highest levels of care-seeking being observed for February through April 2021 and the lowest for November 2020 through January 2021 (Table 3).

Given the small size of the subsample with a history of COVID-19, we were not able to run adjusted regression models to identify factors independently associated with having sought care for COVID-19.

### COVID-19 vaccination

Vaccine questions were added to the survey in late January 2021. Overall, 70.1% of migrants had received at least one dose of vaccine against COVID-19. Among them, 99.9% reported having received the vaccine in the U.S. and 0.2% in Mexico. Bivariate analyses revealed significant associations between the likelihood of having received the vaccine and older age (Linear-by-linear Association = 5.71, *p* = 0.017), English fluency (Likelihood Ratio =8.52, *p* = 0.037), time in the U.S. (Likelihood Ratio =21.65, *p* < 0.001), greater time in detention (Linear-by-linear Association =6.00, *p* = 0.014), being detained in a place other than an immigration detention center (Chi square =4.93, *p* = 0.026), immigration status (Fisher's Exact Test, *p* = 0.049), survey location (Likelihood Ratio =12.99, *p* = 0.002), and survey quarter (Likelihood Ratio =24.11, *p* < 0.001).

After adjusting for demographics, age, English Fluency, time living in the U.S., time in detention, type of detention facility, and survey city remained significantly associated with vaccine receipt. No regression models could be estimated for history of migration to the U.S. or survey quarter due to the lack of variation in vaccination status for some categories of these independent variables (Table 4).

**Figure 1:**
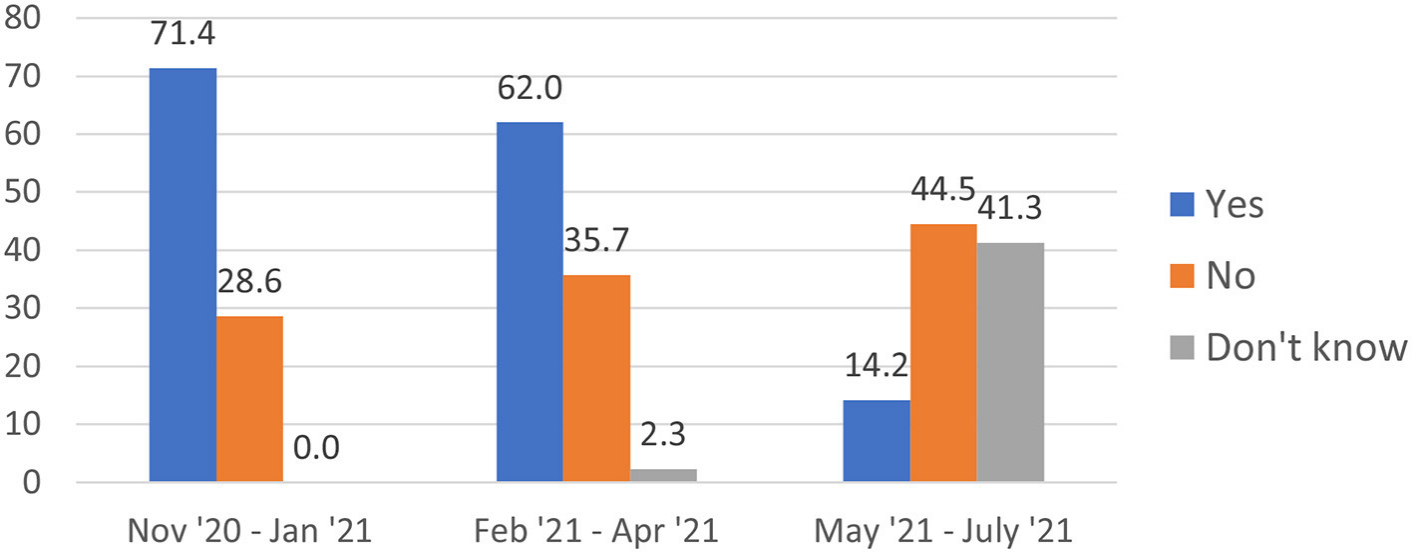
Intentions to get vaccinated against COVID-19 among non-vaccinated deported migrants by survey quarter (%).

Among the 29.9% of migrants who had not yet been vaccinated, 41% reported no intention of getting vaccinated, 32.5% intended to take the vaccine, and 26.5% did not know if they would get vaccinated. Reasons for vaccine hesitancy among those who did not intend to get vaccinated or did not know if they would included concern about potential side effects of the vaccine (74.0%), not knowing where to obtain the vaccine (13.0%), not believing in the efficacy of the vaccine (4.8%), concern about data collected at vaccine sites (2.0%), mistrust of doctors (0.6%), and other (not specified) reasons (2.4%). Over time, the percent of unvaccinated migrants who intended to get vaccinated decreased from 71.4% in the second quarter of the survey to 62.0% in the third quarter and 14.2% in the last quarter of the survey. Inversely, from the second to the last quarter, the percent of migrants who did not plan to get vaccinated increased from 28.6 to 44.5%, and the percent who did not know if they would get vaccinated increased from 0 to 41.3% throughout this period (Figure 1).

### Other preventive and risk factors

Regarding other preventive measures, 98.1% of migrants reported wearing masks and 96.3% washing or sanitizing hands frequently. In contrast, 62.6% reported staying home most of the time and a mere 0.4% were able to work from home. Importantly, an estimated 44.3% of migrants reported having been confined in crowded or small spaces with too many other detainees while in detention in the U.S. (Table 2).

## Discussion

This study sought to reduce the gap in knowledge regarding the impact of the COVID-19 pandemic on migrant populations and, more specifically, on deported Mexican migrants. The findings indicate that although most migrants had been tested for COVID-19, about 15% of them had not been screened for the infection before they were deported by U.S. immigration authorities at the border or before they were released by Mexican immigration authorities to the larger community in the Mexican border region. This figure is consistent with previous media reports denouncing that migrants in Border Patrol custody were not tested unless they showed symptoms (37). However, those reports also indicated that “all [migrants] are tested when they leave Border Patrol custody,” a claim that stands in stark contrast to results from our survey, which indicate that a sizable subset of this population had not been tested at any point since the start of the pandemic. Testing rates improved over the course of our survey, probably owning to improvement in availability of testing supplies and services as the pandemic evolved. Yet, the suboptimal testing levels among this transnational population, who are subject to detention in congregate settings, illustrates some of the mechanisms through which immigration enforcement policies can facilitate transmission of COVID-19 across international borders: migrants are arrested, kept in crowded detention facilities, and deported without first being screened for COVID-19.

This study also found that about one in three migrants had been diagnosed with, tested positive for, or suspected they had had COVID-19 infection, with prevalence rates increasing over time. In the context of insufficient testing and low levels of access to healthcare, this figure is likely an underestimate of the true burden of the pandemic on this population. It is important to note that the risk of infection was associated with migration-related experiences. Migrants detained for over a year or detained in prisons or jails had a higher risk of infection than those detained for brief periods of time or in immigration centers. It is well-established that congregate settings where large numbers of people are gathered in close proximity or for extended periods of time can facilitate transmission of respiratory infections, including COVID-19. Prisons and immigration detention centers present limited options for practicing COVID-19 preventive measures, such as social distancing (38, 39). Our survey reveals that over 44% of deported migrants were held in crowded spaces while in detention in the U.S. Together, these findings suggest that detention for long periods of time in the U.S. contributed significantly to COVID-19 exposure and transmission among deported Mexican migrants, especially those detained in prisons or jails vs. immigration detention centers. This study also indicates that insufficient measures were put in place to protect detained migrants from COVID-19 in detention facilities. Surprisingly, authorized migrants and those with higher education were more likely to report a history of COVID-19 than their unauthorized or less educated counterparts. This could reflect true higher infection rates among these groups, but these associations may also be indicative of authorized and more educated migrants facing fewer barriers to accessing testing and diagnostic services. Over time, the odds of having a COVID-19 infection history changed significantly and independently from other factors, with a marked decreased in infection odds from November 2020 through January 2021 and a spike from February to April 2021, compared to the first quarter of the survey. These variations may be related to changes in pandemic-related travel restrictions and other control measures imposed by U.S. and Mexican authorities at different points during the pandemic.

One of the most striking findings from this study is the fact that more than one in three migrants diagnosed with or suspected of having COVID-19 never sought medical attention for a disease that can be potentially deadly. While the small sample size precluded estimation of adjusted regression models, our bivariate analyses suggest greater odds of seeking COVID-19 treatment among deported migrants who were detained for longer periods of time compared to those who were in detention for less time. This is consistent with other surveys of unauthorized Latino immigrants that have found that, in general, this population's access to COVID-19 testing and treatment (40) in the U.S. is severely restricted. Fear of deportation (11, 41), concerns regarding future regularization, limited English fluency, lack of health insurance, and lack of knowledge regarding where to obtain health care services have been extensively documented as barriers to health care services among Latino migrants. Considering that U.S. prisons are known to have inadequate prevention and treatment services for incarcerated persons (42), this result is even more troubling and calls for implementation of efforts to improve access to COVID-19 treatment and adequate health care, among migrants both in detention facilities and in the community at-large.

Vaccination rates, at about 70%, were higher than expected among this population and they increased over time as the survey progressed. Shortly after the completion of our survey, by September of 2021, the CDC was reporting that 74.4% of U.S. adults had received at least one dose of the COVID-19 vaccine, including 71.5% of all Latinos. Despite the frequent allusions to vaccine hesitancy as a driving factor for low vaccination levels among racial and ethnic minorities in the U.S. (43), research indicates that language barriers, fear of deportation, and lack of access to vaccination sites are more important barriers to vaccination among migrants in the U.S (44). The relatively high immunization rates found by our survey and the sizable fraction of migrants not yet vaccinated who intended to seek a vaccine, are further evidence that when given the chance, most migrants will accept the opportunity to get vaccinated to reduce their risk of COVID-19. These better-than-expected vaccination levels are also consistent with trends observed for Latinos in the U.S., who have achieved higher vaccination levels than their non-Hispanic counterparts, despite having had a slower uptake during the initial phases of the vaccine roll-out (45). As it was the case for testing, the odds of vaccination were also lower among migrants detained in immigration centers compared to migrants detained in other types of facilities, which underscores a missed opportunity to provide these underserved populations with preventive services while in ICE-operated detention settings. For the subset of deported migrants who did not plan to get vaccinated, our findings show that concern about side effects and not knowing were to obtain the vaccine were the most frequent barriers. This data calls for deployment of outreach and education programs addressing these barriers to further augment Latino migrants' immunization levels.

Our analyses also revealed that other demographic and migration variables were significant, independent determinants of COVID-19 testing, infection, care seeking, and vaccination among deported migrants. For example, older migrants were more likely to be tested and vaccinated, possibly due to greater perceived severity of COVID-19 infection and earlier eligibility for vaccination among older individuals compared to their younger counterparts. Not married migrants were more likely to be tested, an intriguing finding that merits more research in the future. As expected, English fluency was an independent predictor of COVID-19 testing and vaccination. This finding is consistent with other research regarding language as a barrier to preventive services in migrant and refugee populations (44). History of migration showed an inconsistent pattern of associations with testing, infection, and vaccination. On the one hand, migrants with a history of migration to the U.S. and those who considered the U.S. their country of residence had higher adjusted odds of being tested than those who had never migrated to the U.S. or still called Mexico their home. However, length of time in the U.S. did not increase the odds of testing, infection, or vaccination rates. In fact, compared to new migrants <2 years of time in the U.S., migrants with 3–10 years of residence in the U.S. showed significantly lower odds of testing, infection, and vaccination, and migrants with 11 or more years in the U.S. also had lower odds of vaccination than newer migrants. These findings could be related to increased acculturative stress with longer time spent in the U.S. (46) and call for additional research to understand the nature of these associations. Our study also revealed significant associations between contextual variables and COVID-19 outcomes. The odds of COVID-19 testing were greater among migrants deported through Matamoros and Ciudad Juarez, perhaps reflecting greater availability in the community and detention settings in the regions that deport migrants through these cities. In contrast, compared to migrants deported through Tijuana, care-seeking and vaccination were less likely to be reported among migrants deported through Matamoros and Ciudad Juarez, respectively. The reasons behind these findings need to be elucidated, but they call for interventions to improve access to testing, care, and vaccination for migrants in catchment areas that fare worse in these domains.

### Limitations

This study has several limitations, including the cross-sectional design that impedes testing for causal associations, an imperfect response rate that creates the potential for self-selection bias, and some age and geographic differences between respondents and non-respondents. In addition, our exclusive reliance on self-reported data creates the potential for recall and social desirability biases. Our findings showed that COVID-19 outcomes among deported migrants varied significantly depending on the city through which they were deported, even after controlling for socio-demographic characteristics. These differences may reflect different levels of risk and access to services for detained migrants across different U.S. regions. Our survey was implemented only in Tijuana, Ciudad Juárez, and Matamoros, and the percent of migrants sampled in Ciudad Juárez was very small compared to the other two cities. Hence, our overall estimates of COVID-19 outcomes may be more representative of migrants deported through Tijuana and Matamoros and less accurate for individuals who are deported through other Mexican border points. We acknowledge that analyzing the samples from the three cities together can complicate interpretation of findings. However, the sample size within each city was small (especially in Ciudad Juárez) and stratified analyses for each city would have yielded even smaller cell counts and less precise estimates. The small sample size and combination of data from three different cities call for caution when interpreting the findings. Finally, the deported population is largely composed of male migrants and, consequently, most of our sample was male. It is important to bear in mind that the findings from this study may be more reflective of the experiences of male migrants than those of their female counterparts. Future research must aim to include larger samples of female deportees.

### Implications for public health practice and research

Overall, the findings from this study underscore the role of detention and deportation as structural risk factors for COVID-19 infection among Latino migrants. They also provide evidence of the failure to deliver effective prevention, testing, and treatment of COVID-19 for this population in both the U.S. and Mexico. Increased risk for COVID-19 adds to the myriad mental and physical health impacts of the civil and human rights abuses experienced by detained migrants in the U.S., the country with the largest immigration detention system (47) in the world. In general, our results resonate with calls from other scholars for decarceration of migrant detainees, suspension of deportations, and the need to identify alternative immigration enforcement practices informed by public health and human rights principles. (48). They also demand a better response by the Mexican government to reduce undiagnosed infection and improve vaccination rates among migrants forcibly returned to Mexico by U.S. immigration authorities, as well as other in-transit migrants and asylum seekers (49). For example, every year, over 100,000 Mexican nationals deported from the U.S. are subject to mandatory processing by Mexican migration authorities at deportation stations along the Mexican border. This system creates a unique opportunity to offer this sizable migrant flow COVID-19 rapid testing, triage for care of infected individuals, and vaccination at the point of return to Mexico, prior to migrants' release into the community. This practice would reduce the risk of undiagnosed disease transmission across borders, increase access to proper care among infected deported migrants, and reduce these migrants' risk of suffering severe COVID-19 infection outcomes.

The pandemic is far from over. We are likely to continue seeing new variants of the COVID-19 virus, including potentially more transmissible and/or deadly ones. As we prepare to respond to the new phases of the pandemic, we need better policies to protect unauthorized immigrants from discriminatory policies that increase their risk of exposure to COVID-19 and limit their ability to access timely testing, obtain proper treatment, and adopt preventive measures, including social distancing, vaccination, and booster vaccination. Although our survey did not collect information on receipt of boosters, current CDC data, not disaggregated by nativity, shows that among fully vaccinated people, only 41.3 of Latinos vs. 58.7% of non-Hispanic whites have received a booster dose. Programs that train and involve Latino community members to provide COVID-19 information in Spanish or an indigenous language can improve trust and uptake of these preventive measures (6, 10, 11, 25). These programs should target both urban and rural areas, where resources for migrant populations are often even more scarce (25). Better workplace conditions, financial supports, healthcare coverage, and vaccine distribution are also necessary to mitigate the impact of future phases of the pandemic on this population (10, 17).

This study includes data collected relatively early in the pandemic. Since then, access to testing, treatment, and vaccines have evolved in the U.S. and Mexico. The findings could be different if more recent data were included. Future research must continue to examine COVID-19 outcomes among deportees and other migrants and identify and address barriers to preventive and treatment services among this population, including reasons for vaccine hesitancy.

Moving forward, it is also critical to improve our surveillance systems to safely collect information that allows for disaggregation of COVID-19 indicators by native vs. foreign-born status. These systems should also evaluate risk stratification at work to allow us to better estimate disparities in the burden of COVID-19 shouldered by migrant and immigrant groups and characterize the drivers of these disparities. During the first year of the pandemic, only 28 U.S. states were reporting COVID-19 mortality by race and ethnicity (2) and, to our knowledge, no state reported cases or vaccination figures by nativity. The limited availability of data impedes formulation of evidence-based policies and programs that could mitigate the impact and spread of COVID-19 (35).

## Conclusion

This study provides insights into the extent of COVID-19 testing, infection, care, and vaccination among Mexican migrants deported from the U.S., an underserved and understudied migrant population. The results show that at least a third had a history of diagnosed or suspected infection, and over 44% were held in crowded conditions. The study also demonstrates insufficient access to testing and care for COVID-19, but higher-than-expected levels of vaccination and willingness to get vaccinated among those not yet immunized. As we prepare for future waves of the pandemic and potentially more transmissible and/or deadly variants, decarceration and other measures aimed at reducing COVID-19 risk and increasing access to preventive services and treatment among detained migrants must be planned and implemented.

## Data availability statement

The raw data supporting the conclusions of this article will be made available by the authors, without undue reservation.

## Ethics statement

The studies involving human participants were reviewed and approved by Drexel University Institutional Review Board. The participants provided their written informed consent to participate in this study.

## Author contributions

APM-D conceived, designed the study, obtained research funding, and directed the implementation of the study with support from co-investigators JG-F, EP, AA-G, ML, XZ, and MR. LB helped to coordinate the study, programmed the study surveys, monitored the data quality, and conducted the data analyses under the supervision of APM-D. CC-S assisted with human subjects protocols, personnel training, and review of the literature. APM-D and CC-S led the writing of the first draft of the manuscript. All authors contributed to the article and approved the submitted version.

## Funding

Research reported in this publication was supported by the Eunice Kennedy Shriver National Institute of Child Health and Human Development of the National Institutes of Health under Award Number R01HD046886.

## Author disclaimer

The content is solely the responsibility of the authors and does not necessarily represent the official views of the National Institutes of Health.

## Conflict of interest

The authors declare that the research was conducted in the absence of any commercial or financial relationships that could be construed as a potential conflict of interest.

## Publisher's note

All claims expressed in this article are solely those of the authors and do not necessarily represent those of their affiliated organizations, or those of the publisher, the editors and the reviewers. Any product that may be evaluated in this article, or claim that may be made by its manufacturer, is not guaranteed or endorsed by the publisher.
